# Social norms, social identities and the COVID‐19 pandemic: Theory and recommendations

**DOI:** 10.1111/spc3.12596

**Published:** 2021-04-10

**Authors:** Fergus G. Neville, Anne Templeton, Joanne R. Smith, Winnifred R. Louis

**Affiliations:** ^1^ School of Management University of St Andrews St Andrews UK; ^2^ School of Philosophy, Psychology and Language Sciences University of Edinburgh Edinburgh UK; ^3^ School of Psychology University of Exeter Exeter UK; ^4^ School of Psychology University of Queensland Brisbane Queensland Australia

## Abstract

Sustained mass behaviour change is needed to tackle the COVID‐19 pandemic, but many of the required changes run contrary to existing social norms (e.g., physical closeness with in‐group members). This paper explains how social norms and social identities are critical to explaining and changing public behaviour. Recommendations are presented for how to harness these social processes to maximise adherence to COVID‐19 public health guidance. Specifically, we recommend that public health messages clearly define who the target group is, are framed as identity‐affirming rather than identity‐contradictory, include complementary injunctive and descriptive social norm information, are delivered by in‐group members and that support is provided to enable the public to perform the requested behaviours.

## 1 INTRODUCTION

There is currently no effective medical cure for COVID‐19, and sufficient vaccination to achieve collective immunity is a long‐term project. This means that rapid mass behaviour change is necessary to tackle the pandemic by ‘flattening the curve’ of infections and preventing medical services from being overwhelmed. Widespread behaviour change may also form the basis of eradication or ‘COVID‐zero’ strategies. This ‘new normal’ includes the adoption of behaviours such as maintaining physical distance from people outside your household, regular handwashing or sanitising, the wearing of face masks, uptake of a vaccine when it is offered, and complying with test and trace guidelines to quell localised flare‐ups. Physical distancing and quarantine rules are particularly challenging due to the impact they have on employment, travel and social interaction.

There are two main approaches that authorities can use to try to change public behaviour. The first is *instrumental compliance* which involves commanding behaviour change and expecting obedience through the fear of punishment. However, this strategy may not produce sustainable behaviour change if people only follow guidance when they are visible to authority, and if the public resist commands which are seen as unreasonable or damaging to the good of their social groups (Bonell et al., 2020; Haslam et al., 2014; Jackson, Posch, et al., 2020; Mooijman et al., 2017; Van Bavel et al., 2020). Such change does not result from the internalisation of new ways of behaving into the self‐concept and depends upon external forces or ‘nudges’ (see Mols et al., 2015).

Instead, this paper will focus on the second approach of *normative compliance*, where the public are persuaded that appeals for protective behaviours benefit their social group and are supported by fellow group members. Interventions may have their greatest effect through the indirect influence of group members rather than the original message (Bond et al., 2012; van Bavel et al., 2020). We therefore argue that the key to mass behaviour change and influence is an understanding of social norms and how these relate to social identities. This paper will discuss what social norms are, how they are linked to social identities, how they shape behaviour and how those in leadership roles can encourage social norm change with reference to the COVID‐19 pandemic.

## 2 SOCIAL NORMS AND SOCIAL IDENTITIES

Social norms are rules or standards for behaviour that serve as guides for people's actions, help create expectations about how others will act and promote greater coordination in social life (Smith, 2020). Norms can operate at many levels, ranging from the personal to the collective. At the personal level, people have beliefs about the kinds of behaviour that they should (or should not) engage in; these are often referred to as personal or moral norms (e.g., Bicchieri, 2006). Similarly, societies have beliefs about behaviours that are prescribed or proscribed (e.g., ‘golden rules’, taboos). At the intermediate level, groups, such as organisations or families, will also be associated with specific norms: beliefs about how group members should behave and do behave.

Norms can emerge during group interaction as group members are exposed to the opinions or observe the actions of fellow group members. In one of the classic studies in social psychology, Sherif (1936) demonstrated that groups spontaneously generate their own norms and frames of reference when making judgements about ambiguous stimuli (i.e., how much a point of light in a darkened room moves). Once established, this norm becomes adopted by individuals as their own personal frame of reference, maintained even when the person is no longer in the physical presence of other group members. Groups can then use these prior frames to make sense of new events (Moscovici, 1988). For example, when information emerged about a novel coronavirus in January 2020, there was a lot of uncertainty about the virus and its impact, and people looked to others within their social groups for guidance on how to react. In the United States, Democrats and Republicans came to view the threat of the virus very differently as they converged on attitudes and beliefs that were seen to be consistent with the norms of each group (Crimston & Silvanathan, 2020; Gollwitzer et al., 2020; Pew Research Center, 2020), with downstream consequences for engagement in protective actions (Allcott et al., 2020). A similar picture was seen in the United Kingdom with groups' divergent sense of trust in science and public health behaviour (Maher et al., 2020).

Conformity researchers argue that influence arises through two processes: informational influence or normative influence (see e.g., Deutsch & Gerard, 1955). Informational influence (i.e., the need to be ‘right’) occurs when people use the attitudes or actions of those in their social groups as cues to the correct behaviour. Normative influence in this narrower sense (i.e., the need to be ‘liked’) occurs when people conform in order to be accepted by others within their social group. Thus, in the absence of clear laws, a person might wear a face mask either because the fact that others are wearing masks convinces them that it is the right thing to do to reduce the risk of transmission (i.e., informational influence) or because they do not want to stand out negatively by not wearing a face mask (i.e., normative influence).

While early psychology studies demonstrated the power of social groups for shaping behaviour, many people (researchers, politicians, lay people) interpreted the findings in a negative way. That is, groups were to be avoided and their influence resisted. It is easy to see why this was the case: many of the classic studies in social psychology, conducted in the aftermath of World War 2 and the Holocaust, were understandably focused on explaining the dynamics of tyranny and how group processes could produce horrific outcomes (e.g., Haney et al., 1973; Milgram, 1963). However, groups themselves are now not regarded as bad or good psychologically per se. Rather, it is the *content* of the groups (e.g., their norms, shared beliefs, values) that shape whether social influence will lead to pro‐social outcomes (e.g., the emergence of mutual aid groups) or anti‐social outcomes (e.g., ignoring test and trace guidelines) (Neville & Reicher, 2020).

A dominant framework for understanding how social norms form, change and operate is the social identity approach (Reicher et al., 2010; Spears, 2021), developed in social identity theory (Tajfel & Turner, 1979) and self‐categorisation theory (Turner et al., 1987; see Hornsey, 2008, for a review). The social identity approach proposes that belonging to social groups provides individuals with a definition of the group (i.e., a social identity), and a description and prescription of what is involved in being a group member (i.e., the group's norms). When an identity is activated or salient, people will define themselves and behave accordingly. For example, we may think of ourselves and act in one way at work, as workers, and another way at home, as family members. However, people do not have one single social identity. Individuals belong to multiple groups, such as family groups, neighbourhoods, workplaces or nations, each with its own set of norms. Because norms are tied to social identities, as social relations and definitions of who ‘we’ are shift, so does what ‘we’ approve of and what ‘we’ do. For example, a Scottish man may be likely to wear a mask when his Scottish identity is salient and the category definition of being Scottish includes protecting community members during the crisis (Scottish Government, 2020). In comparison, if his gender identity is salient then it could reduce mask‐wearing due to norms of masculinity (Capraro & Barcelo, 2020; Levita, 2020). Thus, one aspect of effective normative influence is to attempt to ensure that the relevant identities whose norms support the behaviour are salient at the time of the decision.

In thinking about social norms, it is important to note that norms have both injunctive elements (i.e., defining what *should* be done) and descriptive elements (i.e., defining what *is* done; see Cialdini et al., 1990, 1991). Injunctive (or prescriptive) norms refer to beliefs about what action is (dis)approved of and motivate conformity primarily because of the social rewards or punishments associated with performance of the behaviour. Descriptive norms describe what is typical or normal behaviour and motivate conformity primarily by providing information about what action is likely to be effective and adaptive in a specific context. Although injunctive and descriptive norms are often aligned, such that what is approved of is typically done (and vice versa; see Eriksson et al., 2015), this is not always the case. Indeed, attempts to change people's behaviour are often prompted by the fact that injunctive norms (e.g., ‘we should not stockpile food’) and perceived descriptive norms (e.g., ‘I've seen empty supermarket shelves on social media’) are not aligned.

In response, behaviour change agents sometimes attempt to communicate that injunctive and descriptive norms are in conflict, in the hope that raising awareness of this inconsistency will motivate people to engage in the desired behaviour (see Cialdini, 2003). However, research has shown that communicating that people are not ‘practising what they preach’ can undermine engagement in the desired behaviour: a so‐called ‘boomerang’ or ‘backlash’ effect (see Smith et al., 2018; Smith & Louis, 2008). For example, if a government message or newspaper article argues that group members should physically distance but is accompanied by images of crowds apparently not complying with this advice, then this is likely to backfire since the descriptive norm (people are not physically distancing) contradicts the injunctive norm (people should physically distance; Steffens, 2020). Moreover, when social norms are aligned such that group members believe others are performing the necessary costly behaviours, free‐rider theories suggest this may lead to slacking (Olson, 1971). However, this may be overcome by making sure norms are defined in collective terms such that people define their identities as members of a social group rather than as individuals (as is often assumed by free‐rider theories).

It is important to note that social norms are not static intra‐psychic entities. Instead, they are the product of intra‐ and inter‐group interaction such that they are continuously debated, regulated and enforced as groups evolve and social context changes (Smith et al., 2015; Thomas et al., 2019). When a group member's beliefs or behaviour deviate from the descriptive or injunctive norms of the group, these can be challenged by fellow group members. This ‘self‐regulation’ or ‘self‐policing’ is more likely to be accepted and effective when it comes from within the group rather than from outgroup members such as those in authority (Hornsey et al., 2002; Reicher & Stott, 2020; Stott et al., 2012).

Overall, then, a large body of research has confirmed the power of social norms to determine the form and direction of group members' attitudes and actions, particularly those individuals strongly attached to the group, across many behavioural domains (see J. R. Smith & Louis, 2009, for a review). Norms provide powerful meaning‐makers for how we should act and feel towards others. However, group members' perceptions of what the norms are can be inaccurate. For example, research has shown that university students consistently over‐estimate both the level of approval for excessive drinking and the level of excessive drinking among their peers, and that students' own drinking levels are influenced by these misperceptions (e.g., Prentice & Miller, 1993). Students then drink more to bring their behaviour into line with the (misperceived) social norm. A cross‐sectional study has shown that students believe they wash their hands more frequently than their classmates in illness prevention contexts, and this peer perception correlates with their own behaviour (Dickie et al., 2018). Of most relevance, a recent survey reported that 92% of people graded their own compliance with COVID‐19 restrictions as greater than the population average (Fancourt et al., 2020).

Research on social norms and behaviour change has shown that it is possible (albeit sometimes difficult) to change behaviour with clear messages that reinforce positive norms and correct misperceptions (see e.g., Tankard & Paluck, 2016; cf., Foxcroft et al., 2015), particularly if those messages invoke a shared social identity (see Reynolds et al., 2015). It is also possible to leverage small changes for the better within the group, by highlighting dynamic or change norms (‘A growing minority are doing x’) which have been shown to create normative influence (Mortensen et al., 2019; Sparkman & Walton, 2017). However, to secure long‐term change, it is important to appeal to people's membership in valued groups and to change individual behaviour through changing social norms. This can be achieved through effective social identity‐based leadership.

## 3 LEADERSHIP AND FOLLOWERSHIP

According to the social identity approach to leadership (see Haslam et al., 2020), leaders gain and maintain influence by shaping social norms: clarifying group members' understanding of what the group does (or does not) stand for, and defining core values, ideals and behaviours. Leaders can do this because they are seen to be representative (or prototypical) of the group (‘they are one of us’): they are seen to embody the attributes that characterise a group and make it distinct from other groups. Leaders are often given leeway to push the group in new directions (e.g., Abrams et al., 2013; Hollander, 1958), particularly if followers perceive that any change is in the best interests of the group (i.e., that leaders are ‘doing it for us’; Steffens et al., 2014). Thus, leaders are in a unique position to change followers' attitudes and actions. Aside from the importance of providing clear and specific guidance about actions to take (see Bonell et al., 2020; Carter et al., 2016), one way that leaders can encourage particular actions is by crafting a sense of who ‘we’ are (e.g., ‘we stay at home to protect our neighbours and the health service’) and embedding these definitions in structures, rituals and practices (e.g., weekly ‘clap for carers’ to thank frontline health workers). For example, Prime Minister of New Zealand, Jacinda Ardern, framed the national category definition to encourage adherence to lockdown measures, collective resilience and mutual aid: ‘For now, I ask that New Zealand does what we do so well. We are a country that is creative, practical, and community minded. We may not have experienced anything like this in our lifetimes, but we know how to rally and we know how to look after one another, and right now what could be more important than that’ (Vignoles et al., in press).

Moreover, a critical task for leaders is to create and facilitate a sense of shared identity. Leaders can do this in the language they use to engage with followers. For example, Steffens and Haslam (2013) found that politicians who won elections used collective pronouns (i.e., ‘we’) two times as often as politicians who lost elections. If invoking a sense of shared identity influences whether one becomes a leader in the first place, it follows that leaders can strengthen their messages and encourage compliance by crafting these messages in ways that invoke group membership and social norms. By doing so, leaders can foster changes in social norms and group behaviour. Indeed, as Haslam (2020) points out, lauded speeches which encouraged behaviour change during the pandemic made by the Queen and Jurgen Klopp (Liverpool FC manager) used collective pronouns every 19 and 22 words, respectively.

However, ‘we‐ness’ is hard to achieve without a sense of trust in leadership. Trust is a key determinant of whether leaders can secure compliance with their policies (Jimenez & Iyer, 2016; Schmelz, 2021) and engender ‘engaged followership’ (Haslam et al., 2020). If leaders are seen to place themselves above the group, it undermines the sense of shared identity that leaders rely on to lead effectively. Group members trust their leader(s) to the extent that they are seen to be ‘one of us’ who are ‘doing it for us’. For example, Angela Merkel, Chancellor of Germany, set the example she wanted her country to follow by self‐isolating after being in contact with someone who tested positive for COVID‐19 (Bennhold & Eddy, 2020).

On the other hand, if leaders are regarded as outside the social group then shared identity is undermined, and trust and therefore influence in shaping public norms are compromised (Haslam, 2020). This point was demonstrated by public reaction to the news that the UK Prime Minister Boris Johnson's chief adviser, Dominic Cummings, broke lockdown restrictions during the height of the crisis. Johnson's refusal to sack his adviser was seen by some to communicate different rules for the public and those in power. This was associated with a drop in public trust in the government to provide accurate information on the pandemic (19% decrease over 6 weeks; Fletcher et al., 2020), and so provided a warrant for people to resist lockdown restrictions (e.g., British Future, 2020; Mahese, 2020). It is important to note, however, that normative influence occurs through shared social identification. Thus, if the public perceived Cummings—and by association the UK government—to be part of their in‐group then this could discourage lockdown adherence as non‐compliance became seen as normative. However, those who saw Cummings and the government as outgroup were *more likely* to comply with restrictions, as his behaviour was regarded as anti‐normative. In this sense Cummings became an ‘anti‐role model’ (Jackson, Bradford, et al., 2020). When respondents were presented with vignettes about deviation from norms related to COVID‐19 guidance by either in‐group members (fellow nationals) or outgroup members (other national groups), it resulted in negative emotions towards the norm deviators in both groups, and support for retributive measures (stronger towards outgroup members) (Van Assche et al., 2020). However, in the United Kingdom at least, the majority of the public were remarkably quick to adhere to protective health guidance that required new normative behaviours (Drury et al. 2020). The need for state enforcement of compliance with guidance appears to have been minimal. Between the 27 March and 14 May 2020, the police arrested only 36 people in London for breaches of the COVID‐19 legislation (Strategy and Governance (Data Office) and MO6 Covid‐19 Briefing Cell, 2020). In a UK survey of compliance with guidance, 92% of people self‐reported that they had stayed 2 m away from other people when outside their home, and 90% were handwashing more often for 20 s (Duffy, 2020). Even accounting for possible social desirability effects in this type of survey (Daoust et al., 2020), overall public willingness to comply with guidance seems high and examples of non‐compliance are generally related to structural inequalities that prohibit people from complying, such as needing to leave home to earn money due to financial instability (Atchison et al., 2020).

## 4 SOCIAL NORMS AND SOCIAL SUPPORT

In addition to promoting health behaviours, being part of groups also has numerous health benefits that can help in times of crises such as the COVID‐19 pandemic. Social identities can protect members from stressful situations and improve well‐being because they provide the basis for social support (Haslam et al., 2005, 2009; Jetten et al., 2012). Facing a common fate in a time of crisis can generate or strengthen a sense of shared identity amongst people, which in turn can lead to increased collective resilience and acts of solidarity (Drury et al, 2016, 2019; Neville et al., 2020; Ntontis et al., 2019). Indeed, we are more likely to provide help to those who are seen as in‐group members (Levine et al., 2005), and so a sense of being ‘all in this together’ can lead to social norms of mutual aid and support. This has been evident during the pandemic. One of the most striking elements of the public's response to COVID‐19 is how communities have collectively coordinated support to facilitate compliance with norms of protective health measures, such as distributing medical resources and food to enable vulnerable people to stay at home (for examples, see Covid‐19 Mutual Aid UK, 2020).

## 5 SOCIAL NORMS AS A RISK FACTOR FOR INFECTION

The high adherence to these new social norms is particularly striking because the COVID‐19 pandemic has required people to go against behaviours that were previously normative, such as being physically co‐present with in‐group members. Maintaining physical distance from our in‐groups—including friends and family—represents a unique challenge because people feel safe and take joy from being close to in‐group members (Alnabulsi & Drury, 2014; Hopkins et al., 2016; Neville et al., 2020; Neville & Reicher, 2011; Novelli et al., 2013; Templeton et al., 2018, 2020). Cruwys et al. (2020) argue that during the COVID‐19 pandemic public health messages should explicitly state the risk associated with being in physical contact with those we care for most and frame the lack of physical contact as an expression of care. Thus, the norms change to keeping away from fellow group members as a way of providing support to the group.

In addition to physical distance, shared group membership can attenuate health risks in other ways such as through reduced disgust and risk perception toward in‐group members (Khazaie & Khan, 2019; Neville et al., 2020; Reicher et al., 2016). Social norm content can also be a catalyst for increased risk taking which may lead to infection. Examples of this from religious festivals include shaving one's head with the same razor as other pilgrims (Hopkins & Reicher, 2017) or being encouraged to bathe in cold polluted waters as part of a ritual (Hopkins et al. 2018). Leadership and public health messages during pandemics must therefore re‐define the normative content of relevant social identities, such that complying with guidance is seen as for the protection and good of the group rather than a violation of pre‐existing social norms.

## 6 POLARISATION AND PROTEST

A key challenge facing norm compliance in the COVID‐19 pandemic is acknowledging and working with existing identity definitions and norms which otherwise might conflict with engagement in new protective health behaviours (Livingstone et al., 2011). Identity definitions which are incongruent with safe behaviours—for example, around burial practices—can provide obstacles to public uptake of safe norms and lead to conflict with the authorities who try to impose the new measures (Stott & Radburn, 2020). Similarly, leaders risk alienating subgroups when they create norms that cannot be followed by all of society.

The ease and maintenance of norm adoption is partially determined by patterns of societal inequality. For example, despite high public willingness to stay home and self‐isolate, guidance such as staying home is difficult for those from low socio‐economic backgrounds who need to leave home for financial reasons (Atchison et al., 2020). Guidance to distance from vulnerable elderly people is particularly difficult for Black, Asian and Minority Ethnic populations to follow because they are more likely to live in multigenerational households (Loftquist, 2012) and in densely populated areas (Jackson et al., 2000). Failure by leaders to address inequalities and provide populations with the support needed to follow the new norms can result in unrest and protest (Drury & Tekin Guven, 2020; Mazepus & van Leeuwan, 2019). Examples of this can be seen in South America (BBC, 2020) where street protests have argued against the lack of financial support from governments which would allow the public to stay home. Moreover, the differential impacts of the pandemic on different social groups could lead to scapegoating and exclusion of already marginalised groups. To mitigate backlash, facilitate compliance with new norms and encourage a shared sense of unity, leaders must provide support systems that allow all groups to adopt and maintain protective norms (see also, Louis et al., 2020).

## 7 RECOMMENDATIONS

We have argued that social norms and social identities are critical to the mass behaviour change necessary to tackle the COVID‐19 pandemic. To summarise, we will finish with five recommendations for how to harness the power of social norms to achieve this goal.

(1) Public health messages should clearly define who the target group is (i.e., what is their social identity, Livingstone, 2020). This should be an identity which is inclusive enough to be relevant to large numbers of people (e.g., the nation) and a social group with which people identify
(2) Behavioural change should be framed as identity‐affirming rather than identity‐contradictory (Livingstone, 2020). The best way of achieving this is often through invoking higher‐order group values (e.g., as a community we look out for and support each other) which then relate to specifically requested behaviours (e.g., physical distancing) (Drury et al., 2020)
(3) Public health messages should include both injunctive and descriptive norm information, and these should not contradict one another (Bonell et al., 2020; Drury et al., 2020). Examples of unwanted group behaviour (e.g., stockpiling of food, failing to physically distance) should not be included as these may unintentionally present a descriptive norm (my group is doing these behaviours) which contradicts the injunctive norm (my group does not approve of these behaviours). Alternatively, this descriptive information could be presented in the reverse direction, e.g., the vast majority of group members are acting in this positive manner
(4) The source of communication is crucial. Messages should come from people who are seen as ‘one of us’ rather than someone from outside (Bonell et al., 2020; Haslam, 2020; Steffens, 2020). This in‐group membership gives credibility to define what is normative for the group and to engage the followership and compliance of fellow group members. Related to this point, behavioural interventions should be co‐designed with group members—rather than imposed by outside forces—such that the community is involved in the development of new norms (Drury et al., 2020; Stott & Radburn, 2020)
(5) Group members must be capable of performing the requested changes in social norms. Authorities must therefore provide scaffolded support systems to enable the public to perform the required behaviours, and these must be seen to be equitable to avoid polarisation and loss of influence (Crimston & Silvanathan, 2020; Stott & Radburn, 2020)
